# Modern Cookery, in All Its Branches, Reduced to a System of Easy Practice for the Use of Private Families; in a Series of Receipts, Which Have Been Strictly Tested, and Are Given with the Most Minute Exactness

**Published:** 1845-10

**Authors:** 


*Modern Cookery, in all its branches, reduced to a system of Easy Practice for the use of private families; in a series of receipts, which have been strictly tested, and are given with the most minute exactness.* By Eliza Acton. *Illustrated with numerous wood cuts. To which are added directions for carving, garnishing, and setting out the table; with a table of weights and meassres; the whole revised aud prepared for American Housekeepers,* by Mrs. S. J. Hale. From the second London edition: small 8vo. pp. 418. Philada., 1845.

It was well said by the facetious author of the Cook's Oracle—himself a physician—that "improvements in agriculture and the breed of cattle have been encouraged by premiums. Those who have obtained them have been hailed as benefactors to society; but the *art* of making use of these means of *ameliorating life,*

*and supporting a healthy existence*—COOKERY—has been neglected. While the cultivators of the raw materials are distinguished and rewarded—the attempt to improve the processes, without which neither vegetable nor animal substances are fit for the food of man, (astonishing to say) has been ridiculed as unworthy the attention of a rational being ! ! !" Yet, we apprehend the ridicule has been confined to a few ; for in all ages the cook has been esteemed a most important personage. In the time of the first Roman Emperors, when the pleasures of the table were carried to the extreme of licentiousness, upwards of 4000 dollars was by no means an uncommon salary for the cook. Mark Antony once presented his cook with a whole *municipium,* solely because he succeeded in dressing a pudding to the satisfaction of Cleopatra ; and Harry the 8th of England parcelled out one of the Crown manors to a lady who had compounded a pudding to his liking. Still, as at the present day, the morality of the cook has often been doubted ; and the elevated *Science de la Gneule* has doubtless been occasionally represented by knaves and rascals,—a fact signalized by the word *coquin,* which bears too many evidences of having been derived from the *cuisine.* "De toutes les étymologies qu'on donne de ce mot"—says Ménage—"celle qui me plaît davantage, et qui me paroît la plus naturelle, c'est celle qui le dérive de *coquus,* cuisinier, ou plutôt de *coquina,* cuisine. *Coquinus* s'est dit originairement des plus bas officiers de cuisine et ensuite des gens les plus vils et les plus méprisables."

Cookery is in reality a part of hygienic medicine. "The stomach,"—as the writer already quoted has remarked—"is the mainspring of our system ; if it be not sufficiently wound up to warm the heart and support the circulation, the whole business of life will in proportion be ineffectively performed—we can neither *think* with precision, *sleep* with tranquillity, *walk* with vigour, nor *sit down* with comfort. There would be no difficulty in proving, that it influences (much more than people in general imagine) all our actions: the destiny of nations has often depended on the more or less laborious digestion of a Prime Minister." And he subsequently adds—"thus does the HEALTH always, and very often the LIFE of invalids, and those who have weak and infirm STOMACHS, depend upon the care and skill of the COOK.

Our forefathers were so sensible of this, that in days of yore, no man of consequence thought of making a day's journey without taking his MAGISTER COQUORUM with him."

We are not to be surprised, then, at the fact, that some of the best cookery books should have been written by physicians. Apicius Cœlius, *De Arte Coquinariâ,* was translated in 1513, with notes by Dr. Martin Lister. In 1658, appeared Archimagirus's Receipts in Cookery, by Sir Theodore Mayerne, Physician to Charles II. Dr. Salmon's Cookery appeared in 1710; Dr. King's Art of Cookery, in verse, in 1740. Mrs. Glasse's Cookery was written by Sir John Hill. In 1807 was published *Culina Famulatrix Medicinæ,* by A. Hunter, M. D., F. R. S.; and last, but not least, came the Cook's Oracle, by the eccentric Kitchener, who affirmed, that he had "not presumed to insert a single composition, without previously obtaining the '*imprimatur*' of an enlightened and indefatigable 'COMMITTEE OF TASTE,' (composed of thoroughbred GRANDS GOURMANDS of the first magnitude;")—although we know it was shrewdly suspected that the Doctor himself constituted the whole committee.

The work before us strikes us as equal—if not superior—to any of its predecessors. Kitchener—in spite of its merits, which are not few or far between—is somewhat *passé*; Mrs. Rundle scarcely retains her elevated position: she was always too *recherché:* Miss Leslie is excellent, but still an opening existed for a *scientific* work on the "culinary art," which was in all respects "up to the day." Such a work, we think, is Miss Acton's; and accordingly we recommend it to the favorable notice of our readers.

